# The Effect of Biopsychosocial-Spiritual Factors on Medication Adherence in Chronic Diseases in Türkiye

**DOI:** 10.1007/s10943-025-02317-3

**Published:** 2025-04-24

**Authors:** Aylin Bilgin, Ayser Doner, Gulyeter Erdogan Yuce, Gamze Muz

**Affiliations:** 1 Internal Medicine Nursing Department, Faculty of Health Sciences, Sakarya University of Applied Sciences, 54400 Sakarya, Turkey; 2 Department of Internal Medicine Nursing, Semra ve Vefa Kucuk Faculty of Health Sciences, Nevsehir Haci Bektas Veli University, Nevsehir, Turkey; 3 Department of Emergency Assistance and Disaster Management, Faculty of Health Sciences, Aksaray University, Aksaray, Turkey

**Keywords:** Anxiety, Chronic disease, Medication adherence, Spirituality, Social support

## Abstract

This study was to examine the factors associated with medication adherence in individuals with chronic diseases within the concept of the biopsychosocial-spiritual model. A cross-sectional study design was used. A total of 837 patients diagnosed with chronic diseases were included in this study between November 2022 and December 2023. Data were collected based on the biopsychosocial-spiritual model. Medication adherence level was evaluated with the “Medication Adherence Report Scale.” “Hospital Anxiety Depression Scale,” “Multidimensional Perceived Social Support Scale,” and “Spiritual Well-Being Scale” were used to evaluate the psychological, social, and spiritual dimensions. Data were analyzed using descriptive statistics and a multiple linear regression model. Anxiety, social support status, and spiritual status were important correlates of medication adherence levels in individuals with chronic diseases. It was determined that variables such as age, gender, and number of chronic diseases did not affect medication adherence. Medication adherence in individuals with chronic diseases is significantly associated with psychological, social, and spiritual factors. Therefore, when evaluating medication adherence, it should not be forgotten that it is a multifaceted concept and individuals should also be evaluated from psychological, social, and spiritual perspectives.

## Introductıon

The increase in life expectancy and the aging of the world population have led to an alarming increase in the frequency of chronic diseases (Boersma et al., 2020; Shi et al., 2021). According to the Centers for Disease Prevention and Control (CDC), chronic diseases are generally defined as conditions that last for one year or longer, require constant medical intervention, or limit daily living activities (Schmidt, 2016). The World Health Organization reports that non-communicable diseases such as cancers, heart diseases, chronic respiratory diseases, strokes, and type 2 diabetes mellitus are responsible for 71% of all deaths globally (Budreviciute et al., 2020). Management of chronic diseases that have significant mortality, morbidity, and multifaceted systemic effects is a very important issue. The main goals in the treatment of chronic diseases are to prevent exacerbations, control symptoms, ensure well-being, prolong life, and improve quality of life (Romeyke et al., 2020). In parallel, medication adherence is of great importance for the course of diseases.

Medication adherence is often defined as the degree to which patients take medications as prescribed by their doctor (Hyvert et al., 2023). This includes factors such as remembering to take medications on time, getting prescriptions filled, and understanding instructions. In case of non-adherence with medication treatment, the prognosis of diseases worsens, and the mortality rate increases. In addition, non-adherence to medication therapy leads to an increase in complications in chronic diseases and an increase in hospitalization rates (Gast & Mathes, 2019).

Studies have reported that individuals with cardiovascular disease experience twice as many complications and have higher mortality rates due to non-adherence to medication treatment (Du et al., 2017; Jüngst et al., 2019). In a study conducted with individuals with chronic diseases, it was emphasized that the adherence rate with medication treatment was 59.1% (Jüngst et al., 2019). Many factors affect medication non-adherence in individuals with chronic diseases (Al-Noumani et al., 2023a, 2023b). Although the main factors are the increase in the number of medications, drug side effects, comorbidities, age, disease perception, and social factors, a multidimensional evaluation is required to better understand the underlying causes of medication non-adherence (Gast & Mathes, 2019; Shahin et al., 2019; Yosef et al., 2023).

## Background

The biopsychosocial model, which provides evaluation with a multidimensional and holistic approach, is a widely used model. The model was introduced by George L. Engel, who was involved in the psychosomatic movement (Matrenitsky, 2021). This model emphasizes the need for a comprehensive evaluation that addresses biological, psychological, and social dimensions to better understand patients and better reveal the consequences of the disease. In recent years, the holistic approach to healthcare has come to the forefront and there has been increased interest in the biopsychosocial model in understanding patients’ experiences or health outcomes (Hatala, 2013). In addition, Sulmasy argued that the spiritual model should be added in order to maintain holistic patient assessment and health services, and with the support of other researchers, a biopsychosocial-spiritual model was created. (Matrenitsky, 2021).

Due to the multifaceted nature of medication adherence, it is a very important issue to evaluate it in a way that includes all factors. Medication adherence level can be associated by biological factors such as the patient’s age, number of comorbid diseases, and number of medications used, as well as psychological factors including depression, anxiety, and stress (Pietrzykowski et al., 2020). Additionally, previous studies emphasize that the treatment and disease adaptation of individuals with chronic diseases is significantly affected by the support they receive from their family or social environment (Fan et al., 2021; Gast & Mathes, 2019; Saffari et al., 2019). Lack of social support is a contributing factor to medication non-adherence (Shahin et al., 2021). Patients with a lack of social support report poorer communication with physicians, lower levels of trust in physicians, lower levels of satisfaction with care, and lower levels of adherence to the medication regimen (Huang et al., 2021). People who receive support from families, friends, and other individuals feel safer and have an optimistic outlook on life. This encourages individuals to comply with treatment (Shahin et al., 2021).

Apart from social support, it has been stated in the literature that the spiritual state of patients in chronic diseases such as asthma, heart failure, and hypertension also affects medication adherence (Elhag et al., 2022; Helvaci et al., 2020). Spirituality is the essence of being and an effort to find an individual’s place in the universe, the meaning of life, and the relationship with other people, the community, and God (Helvaci et al., 2020; Koenig & Carey, 2025). Individuals with chronic diseases turn to spiritual practices to cope with situations such as symptom burden, hospitalizations, treatment process, stress, and anxiety (Badanta-Romero et al., 2018). These practices enable patients to accept the disease, increase faith in treatment, find meaning in life, recuperate, reduce stress, and increase adaptation to changes. This positive coping process increases participation and adherence with the treatment process (Shahin et al., 2021).

Considering the increasing frequency of chronic diseases and the multifaceted nature of medication adherence, it is important to evaluate medication adherence and affecting factors with a holistic approach. However, no study has been found in the literature that examines medication adherence in chronic diseases in depth within the scope of the biopsychosocial-spiritual model. This study may contribute to a better understanding of medication adherence and related factors based on the biopsychosocial-spiritual model and to planning appropriate education and counseling based on the needs of patients. This study is a descriptive, cross-sectional study that aims to examine the factors related to medication adherence in chronic diseases within the scope of the biopsychosocial-spiritual model.

Research questions in this study

1. What is the relationship of biological factors such as age, gender, number of chronic diseases with the medication adherence level of individuals with chronic diseases?
2. What is the relationship of anxiety and depression, which are psychological factors, with the medication adherence level of individuals with chronic diseases?
3. What is the relationship of multidimensional social support, one of the social factors, with medication adherence levels of individuals with chronic diseases?
4. What is the relationship of spiritual well-being, one of the spiritual factors, with the medication adherence level of individuals with chronic diseases?

## Methods

### Study Design and Setting

This study is a descriptive, cross-sectional type of study and was conducted as a nationwide online survey. An anonymous online survey was conducted on the Google Forms survey platform and distributed using online platforms (phone, text, e-mail, social media, etc.). Data were collected in Türkiye in November 2022–December 2023. The study was conducted and reported according to the Strengthening the Reporting of Observational studies in Epidemiology (STROBE) checklist (Vandenbroucke et al., 2021).

### Participants

The study is a survey study to be conducted across the country. For this reason, the snowball sampling method was used to reach a wide sample and create a rich source of information. In the snowball sampling method, individuals who were suitable for purpose of the study and met the inclusion criteria were identified through online platforms (phone, message, e-mail, social media, etc.), and individuals who could convey this were selected as a starting point, and if the people reached suggested other names, the snowball continued to grow. The first participant was reached from the researcher’s social network via WhatsApp, and the sample grew as the first participant suggested another participant.

**Fig. 1:**
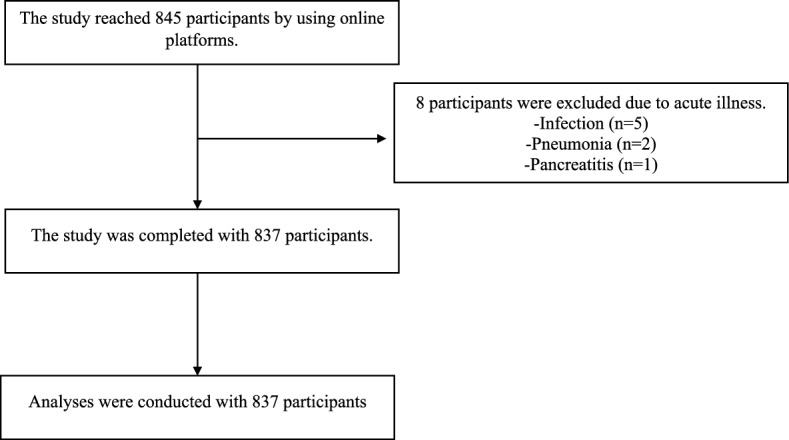
Flow diagram of the study sample

Sample size was calculated using G*Power 3.1.9.4 software (Faul et al., 2009). In the calculation, using the linear regression fixed model, the minimum sample size to be included in the study was found to be 759 patients with effect size = 0.02, α = 0.05, and 80% power. Within the scope of the study, 845 participants were reached, but since only chronic diseases were included in the study, individuals with acute diseases were excluded before data analysis. Eight participants were excluded due to infection, pneumonia, or pancreatitis. The study was completed with 837 participants (Fig. 1).

*Criteria for inclusion in the study:* (1) Receiving medical treatment for at least one chronic disease, (2) having the ability to read and understand Turkish, (3) smartphone, computer, etc., can use and access the online survey via smartphone or computer, (4) patients with at least primary education level. *Criteria for exclusion from the study:* (1) Does not have any chronic disease, (2) those with cognitive and psychiatric diagnoses (schizophrenia, dementia, Parkinson’s, and Alzheimer’s, etc.), (3) having problems communicating, (4) smartphone, computer, etc., and cannot access the online survey via smartphone or computer, (5) illiterate. *Exclusion criteria after the study begins:* (1) Participants who answer the questions incompletely in the online survey will be excluded from the study.

### Measurement Tools

This study was conducted based on the biopsychosocial-spiritual model, which is frequently used in evaluation with a holistic approach. In this study, all components of the model were taken into account and factors affecting medication adherence were evaluated with a holistic approach. In this regard, factors such as age, gender, and number of chronic diseases included in the participant information form were examined to evaluate the biological aspect. “Hospital Anxiety Depression Scale” was used to reveal the psychological dimension of the model. “Multidimensional Perceived Social Support Scale” was used to evaluate the social dimension. Finally, the "Spiritual Well-Being Scale" was used to evaluate the spiritual dimension. This study aimed to determine the biological, psychological, social, and spiritual factors that affect medication adherence. Therefore, the dependent variable of this study is medication adherence; the independent variables were biological, psychological, social, and spiritual factors.

*Participant Information Form:* This form was created by relevant literature and researchers (Jüngst et al., 2019; Seng et al., 2020). The form includes variables such as age, gender, education level, marital status, employment status, smoking status, alcohol use status, chronic diseases, number of medications used daily, and hospitalization status in the last year.

*Hospital Anxiety Depression Scale (HADS):* This scale was developed by Zigmond and Snaith (1983) and consists of a total of 14 items. Odd numbered items in the scale reveal anxiety and even numbered items reveal depression (Zigmond & Snaith, 1983). The scale is a four-point Likert type, and the scores of the items vary between 0 and 3. The Cronbach’s alpha value was found to be 0.84 for anxiety, 0.79 for depression, and 0.88 for the total score (Spinhoven et al., 1997). The validity and reliability of the scale for the Turkish population were determined by Aydemir et al., and Cronbach’s alpha coefficient was determined as 0.85 for the anxiety subscale and 0.77 for the depression dimension (Aydemir et al., 1997). In this study, Cronbach’s alpha coefficient was determined as 0.82 for the anxiety subscale and 0.76 for the depression subscale.

*The Multidimensional Scale of Perceived Social Support (MSPSS):* MSPSS was developed by Zimet et al. and consists of 12 items (Zimet et al., 1988). The scale has three subscales: family, friends, and a special person. The lowest score to be obtained from the scale is 12, and the highest score is 84. The Cronbach’s alpha value of the original scale was found to be 0.88 (Zimet et al., 1988). The Turkish validity and reliability study founded by Eker and Arkar, and Cronbach’s alpha value was reported to be 0.86 (Eker et al., 2001). In this study, Cronbach’s alpha value was 0.93.

*Spiritual Well-Being Scale:* The original scale was developed by Peterman et al. and consists of 12 items. The scale has three subdimensions: meaning, peace, and faith. The lowest score that can be obtained from each subscale is 0, and the highest score is 16, while the total scale score ranges from 0 to 48. A high score generally means high spiritual well-being. The Cronbach alpha value of the original scale was found to be 0.87 (Peterman et al., 2002). Turkish validity and reliability study of the scale, Cronbach’s alpha value was found to be 0.87 (Aktürk et al., 2017). In this study, Cronbach’s alpha value was 0.85. There are however restrictioins in using this scale which are noted in the limitations section.

*Medication Adherence Report Scale (MARS): It* is a scale developed by Horne and Hankins to evaluate medication adherence (Horne & Weinman, 1999). Scores from the scale vary between 5 and 25. The Cronbach alpha value of the original scale was found to be 0.85 (Horne & Weinman, 1999). Turkish validity and reliability of the scale were conducted by Temeloğlu Şen et al., and the Cronbach alpha value was determined as 0.78 (Temeloğlu Şen et al., 2019). In this study, Cronbach’s alpha value was found to be 0.91.

### Data Collection

The data were collected online to reach more individuals diagnosed with chronic diseases across the country. An anonymous online survey was conducted on the Google Forms survey platform. While preparing the online survey, the recommendations of the Association of Internet Researchers (AoIR) Ethics Study Committee in Internet research were used (Stommel & Rijk, 2021). Participants were able to see study questions after reading the consent form and clicking the “I have read the information and agree to participate in the study of my own free will” button.

Participants were given an online informed consent form, which included information about the purpose of the study, the importance of answering the questions sincerely and honestly, that the information would be kept confidential, and that they could withdraw from the study at any time. No personal information (phone number, name-surname, e-mail, etc.) was obtained from the participants to ensure anonymity and data confidentiality in the study. Participants who approved the informed consent form filled out the documents sent via “google forms” and finally completed the study with the send button. In order to prevent missing data, all questions in the questionnaire form were requested to be answered, and individuals who did not complete all questions could not send their responses.

### Statistical Analysis

Statistical Package for the Social Sciences (SPSS) 25.0 (Chicago, IL, USA) and Analysis of Moment Structures (AMOS) V24 (Chicago, IL, USA) statistical program were used for the data of the study. percentages. Skewness and kurtosis values were measured to assess the normality of the data. In this study, the skewness values ranged between − 0.812 and 0.978, while the kurtosis values ranged between − 1.225 and 0.850. The data were considered to be normally distributed because both the skewness and kurtosis values were between − 1.5 and 1.5 (Tabachnick et al., 2013). Parametric tests were used depending on the normality of the data. (Independent samples t test, One-way ANOVA test, post hoc test Bonferroni). Percentage, frequency, minimum–maximum values, mean, and standard deviation were used in the study. Pearson correlation test was used to compare the quantitative data and scale results.

A multiple linear regression model was employed to determine the extent to which independent variables influenced medication adherence. The independent variables included age, gender, number of chronic diseases, number of medications used, anxiety, depression, social support, and subdimensions of spiritual well-being (meaning, peace, and faith). The variance inflation factor (VIF) and tolerance values were examined to assess multicollinearity among the predictors. The significance level was set at *p* < 0.05 for all statistical tests. The model’s overall explanatory power was evaluated using the coefficient of determination (*R*^2^), and the statistical significance of the regression model was assessed with an F-test. Individual predictors were examined using unstandardized and standardized regression coefficients (β), t-values, and 95% confidence intervals.

### Ethical Considerations

This study was approved by the XX University Non-Interventional Clinical Research Ethics Committee (Decision Number: 2022/86). The informed consent form containing study details was included on the first page of the survey, and participants who did not agree to participate in the study could not access the questions. These documents will be kept as encrypted files on the researchers’ personal computers. When necessary, data transfer will be provided only between researchers using a personal USB memory stick. The data will not be used for any purpose other than study (lessons, presentations, etc.). Permission was obtained from the relevant author to use the scales in this study.

## Results

The mean age of participants was 48.69 ± 16.52 years old (range 18–91), 65.6% were female, 72.9% were single, 44.0% were primary education level, 42.4% unemployed, and were 50.3% non-smokers. Hypertension was the first among chronic diseases, with 40.5%, followed by diabetes mellitus, with 30.0%. One or three medications daily were being taken by 57.5% of the participants (Table 1). The mean scores were determined as follows: 19.66 ± 4.67 for medication adherence, 53.19 ± 16.14 for social support, 26.22 ± 8.41 for spiritual well-being (8.46 ± 2.72 for meaning, 7.97 ± 2.84 for peace, and 9.78 ± 4.20 for faith), 9.19 ± 4.43 for anxiety, and 8.95 ± 4.37 for depression (Table 2).

**Table 1:** Sociodemographic and clinical characteristics of the participants (N = 837)

Sociodemographic and clinical characteristics		Mean ± SD or n (%)
Age (years)		48.69 ± 16.52
Sex	Male	289 (34.5)
	Female	548 (65.6)
Marital status	Married	227 (27.1)
	Single	610 (72.9)
Education	Primary	368 (44.0)
	High school	146 (17.4)
	Associate degree	66 (7.9)
	Undergraduate	186 (22.2)
	Postgraduate degree	71 (8.5)
Working status	Worker	302 (36.1)
	Unemployed	355 (42.4)
	Retired	180 (21.5)
Smoking status	Smoker	222 (26.5)
	Non-smoker	421 (50.3)
	Ex-smoker	194 (23.2)
Chronic Diseases ^ɸ^	Hypertension	339 (40.5)
	Diabetes mellitus	251 (30.0)
	Hyperlipidemia	167 (20.0)
	COPD/Asthma	188 (22.5)
	Heart failure	78 (9.3)
	Heart rhythm disturbance	117 (14.0)
	Heart valve disease	51 (6.1)
	Coronary artery disease	58 (6.9)
	Anemia	188 (22.5)
	Gastritis/Stomach ulcer	169 (20.2)
	Kidney failure	131 (15.7)
	Other urinary system disease	51 (6.1)
	Thyroid disease	137 (16.4)
	Osteoporosis	58 (6.9)
	Allergy	172 (20.6)
	Others^ʠ^	193 (23.0)
Number of drugs used per day	One or three drugs	481(57.5)
	Four or six drugs	198 (23.7)
	More than six drugs	158 (18.9)
Hospitalization situation	Yes	328 (39.2)
	No	509 (60.8)

M, mean; SD, standard deviation

^ɸ^More than one option has been marked. ^ʠ^ Fibromyalgia, scleroderma, rheumatoid arthritis, inflammatory bowel disease

**Table 2:** Mean medication adherence, social support, spiritual well-being, anxiety, and depression scores of the participants (N = 837)

Variables	Mean ± SD	Min–Max
Medication adherence	19.66 ± 4.67	5.00–25.00
Social support	53.19 ± 16.14	11.00–77.00
Anxiety	9.19 ± 4.43	0.00–21.00
Depression	8.95 ± 4.37	0.00–20.00
*Spiritual well-being*		
Meaning	8.46 ± 2.72	0.00–16.00
Peace	7.97 ± 2.84	0.00–16.00
Faith	9.78 ± 4.20	0.00–16.00

M, mean; SD, standard deviation

Table 3 compares medication adherence, social support, spiritual well-being, anxiety, and depression across sociodemographic and clinical characteristics. It was determined that the medication adherence score differed significantly according to working status (*p* = 0.005), with unemployed participants reporting higher adherence than those who were employed. Social support scores showed a significant difference based on marital status (*p* < 0.001), with single individuals reporting higher perceived social support than married individuals. Regarding spiritual well-being, significant differences were found in subdimensions. Meaning and peace scores were highest among those with a postgraduate degree and lowest among those with primary education (*p* = 0.002, *p* = 0.006). In contrast, in the faith subscale, individuals with a primary education level reported the highest scores compared to those with higher education (p < 0.001). The number of medications used per day was also a significant factor, with those using more than six medications reporting the lowest meaning and peace scores (*p* < 0.001). Hospitalization history was significantly associated with meaning and peace, with participants who had never been hospitalized having higher meaning and peace scores (*p* = 0.019, *p* = 0.046). Female reported significantly higher faith scores compared to male (*p* = 0.046). Anxiety scores significantly varied according to sex (*p* = 0.02), marital status (*p* = 0.003), education level (*p* < 0.001), employment status (*p* = 0.01), number of medications used per day (*p* < 0.001), and hospitalization history (*p* = 0.02). Anxiety levels were higher among female, married individuals, and those who had been hospitalized. Additionally, university graduates had lower anxiety scores than individuals with primary school, high school, or associate degrees, and participants using fewer medications per day (one to three) had lower anxiety than other groups. Employed individuals also had lower anxiety levels than unemployed individuals. Depression scores showed significant differences based on sex (*p* = 0.02), education level (*p* = 0.011), employment status (*p* < 0.001), number of medications used daily (*p* < 0.001), and hospitalization history (*p* < 0.001). Depression was higher in male and those who had been hospitalized. Additionally, university graduates had lower depression scores than associate degree holders, employees, unemployed individuals, and retirees, and participants using fewer medications per day (one to three) had lower depression scores than other groups (Table 3).

**Table 3:** Comparison of medication compliance, social support, mental well-being, anxiety, and depression according to sociodemographic and clinical characteristics (N = 837)

Sociodemographic and clinical characteristics		Medication adherence		Social support			Spiritual well-being						Anxiety		Depression	
							Meaning		Peace		Faith					
		Mean ± SD	*p*	Mean ± SD		*p*	Mean ± SD	*p*	Mean ± SD	*p*	Mean ± SD	*p*	Mean ± SD	*p*	Mean ± SD	*p*
Sex ^†^	Female	19.62 ± 4.73	0.717	53.88 ± 16.13		0.09	8.53 ± 2.74	0.356	8.02 ± 2.85	0.485	9.99 ± 4.23	**0.046**	9.44 ± 4.32	**0.02**	8.70 ± 4.41	**0.02**
	Male	19.74 ± 4.54		51.89 ± 16.13			8.34 ± 2.68		7.87 ± 2.83		9.38 ± 4.14		8.71. ± 4.59		9.42 ± 4.27	
Marital status ^†^	Married	19.18 ± 4.70	0.07	49.84 ± 16.56		** < 0.001**	8.24 ± 2.85	0.142	7.79 ± 2.77	0.272	9.12 ± 4.18	**0.006**	9.93 ± 4.27	**0.003**	8.88 ± 4.45	0.77
	Single	19.84 ± 4.64		54.44 ± 15.82			8.55 ± 2.67		8.03 ± 2.87		10.02 ± 4.19		8.91 ± 4.45		8.98 ± 4.34	
Education ^‡^	Primary (a)	19.95 ± 4.61	0.572	52.05 ± 16.39		0.424	8.10 ± 2.67	**0.002** e > a	7.80 ± 2.76	**0.006** e > c	10.53 ± 3.96	** < 0.001** a > b,d,e	9.50 ± 4.77	** < 0.001** a > b, d, e b, c > d	10.20 ± 4.17	**0.011** c > d
	High School (b)	19.32 ± 5.02		53.76 ± 16.17			8.58 ± 2.69		7.73 ± 2.73		9.16 ± 3.94		9.00 ± 4.12		8.62 ± 4.37	
	Associate’s degree (c)	19.69 ± 4.07		53.18 ± 17.56			8.46 ± 3.23		7.40 ± 3.56		9.87 ± 4.34		10.45 ± 4.50		9.10 ± 3.96	
	Undergraduate (d)	19.44 ± 4.77		54.32 ± 15.07			8.77 ± 2.77		8.36 ± 2.86		9.36 ± 4.45		8.49 ± 3.91		7.11 ± 4.22	
	Postgraduate degree (e)	19.36 ± 4.46		55.05 ± 16.21			9.35 ± 2.16		8.80 ± 2.42		8.14 ± 4.49		8.66 ± 4.10		7.87 ± 4.15	
Working status ^‡^	Worker (a)	18.98 ± 4.70	**0.005** b > a	53.82 ± 16.22		0.599	8.72 ± 2.62	0.110	8.16 ± 2.87	0.305	9.34 ± 4.16	** < 0.001** b > a,c	8.71 ± 4.09	**0.010** b > a	7.64 ± 4.31	** < 0.001** b,c > a
	Unemployed (b)	20.15 ± 4.57		53.13 ± 16.25			8.36 ± 2.70		7.83 ± 2.75		10.55 ± 4.15		9.72 ± 4.49		9.65 ± 4.24	
	Retired (c)	19.83 ± 4.68		52.28 ± 15.83			8.23 ± 2.89		7.90 ± 2.95		8.98 ± 4.14		8.95 ± 4.74		9.80 ± 4.23	
Number of drugs used per day ^‡^	One or three drugs(a)	19.43 ± 4.69	0.251	53.68 ± 16.32		0.477	8.72 ± 2.68	** < 0.001** a,b > c	8.18 ± 2.85	** < 0.001** a,b > c	9.93 ± 4.20	0.267	8.71 ± 4.27	**0.001** b, c > a	7.99 ± 4.18	** < 0.001** c > b > a
	Four or six drugs (b)	19.93 ± 4.40		53.05 ± 15.56			8.73 ± 2.43		8.20 ± 2.58		9.78 ± 3.95		9.67 ± 4.13		9.35 ± 4.02	
	More than six drugs (c)	20.02 ± 4.91		51.89 ± 16.36			7.35 ± 2.92		7.02 ± 2.96		9.31 ± 4.50		10.05 ± 5.05		11.38 ± 4.38	
Hospitalization situation ^†^	Yes	20.05 ± 4.52	0.05	54.09 ± 15.83		0.19	8.19 ± 2.83	**0.019**	7.72 ± 2.88	**0.046**	9.90 ± 4.36	0.503	9.65 ± 4.58	**0.02**	9.86 ± 4.15	** < 0.001**
	No	19.41 ± 4.74		52.62 ± 16.33			8.64 ± 2.64		8.12 ± 2.80		9.70 ± 4.10		8.89 ± 4.31		8.37 ± 4.42	

Bold indicates *p* < 0.05 statistical significance

M, mean; SD, standard deviation

^†^ Independent samples t test ^‡^ One-way ANOVA test, post hoc test Bonferroni

Medication adherence was found to exhibit a weak positive correlation with social support (r = 0.237, *p* < 0.01) and spiritual well-being total (r = 0.340, *p* < 0.01). Among the subdimensions of spiritual well-being, faith (r = 0.324, *p* < 0.01), peace (r = 0.270, *p* < 0.01), and meaning (r = 0.269, *p* < 0.01) all showed weak positive correlations with medication adherence. Conversely, medication adherence displayed a weak negative correlation with anxiety (r = − 0.254, *p* < 0.01) and depression (r = − 0.218, p < 0.01). Additionally, the number of chronic diseases and age were not significantly correlated with medication adherence (*p* > 0.05). Among the other significant relationships, social support was negatively correlated with anxiety (r = − 0.290, *p* < 0.01) and depression (r = -0.398, p < 0.01), while showing a positive correlation with spiritual well-being (r = 0.382, *p* < 0.01). The subdimensions of spiritual well-being—meaning, peace, and faith—were all negatively correlated with anxiety and depression but positively correlated with each other and with social support (*p* < 0.01) (Table 4).

**Table 4:** Correlations between medication adherence, social support, spiritual well-being, anxiety, and depression (N = 837)

Variables		1	2	3	4	5	6	7	8	9
1.	Age	1								
2.	Number of chronic diseases	0.291^**^	1							
3.	Medication adherence	0.055	− 0.022	1						
4.	Anxiety	− 0.046	0.163^**^	− 0.254^**^	1					
5.	Depression	0.247^**^	0.266^**^	− 0.218^**^	0.553^**^	1				
6.	Social support	− 0.050	− 0.188^**^	0.237^**^	− 0.290^**^	− 0.398^**^	1			
7.	Meaning	− 0.049	− 0.196^**^	0.269^**^	− 0.397^**^	− 0.485^**^	0.337^**^	1		
8.	Peace	− 0.015	− 0.193^**^	0.270^**^	− 0.457^**^	− 0.517^**^	0.356^**^	0.768^**^	1	
9.	Faith	0.126^**^	− 0.029	0.324^**^	− 0.304^**^	− 0.250^**^	0.304^**^	0.541^**^	0.553^**^	1

^**^ Correlation is significant at 0.01

In the study, the contributions of biological factors, social support, spiritual well-being, anxiety, and depression on medication adherence were examined, and the overall relationship of the independent variables on medication adherence was found to be statistically significant (F = 15.577, *p* < 0.001, *R*^2^ = 0.148**).** According to the regression analysis results, neither age nor gender had a significant predictor on medication adherence (*p* = 0.824 and *p* = 0.429**)**. Similarly, the number of chronic diseases individuals had was not significantly associated with medication adherence (*p* = 0.622). On the other hand, individuals taking four or more medications exhibited significantly higher medication adherence (β = 0.970, *p* = 0.006). When examining psychological factors, anxiety was negatively associated with medication adherence (β = − 0.123, *p* = 0.004), whereas depression did not have a statistically significant relationship (p = 0.160). Social support emerged as an important factor positively influencing medication adherence, showing a significant positive relationship (β = 0.029, *p* = 0.005**).** Regarding the subdimensions of spiritual well-being, meaning and peace were not significantly associated with medication adherence (*p* = 0.280 and *p* = 0.920). However, faith was identified as one of the strongest predictors of medication adherence (β = 0.247, *p* < 0.001**).** An examination of multicollinearity revealed that tolerance and VIF values were within acceptable limits (Table 5).

**Table 5:** Regression coefficients examining the impact of biological factors, social support, spiritual well-being, anxiety, and depression on medication adherence (N = 837)

Independent variables		Medication adherence									F	*p*	*R*^2^
		Unstandardized Coefficients		Standardized Coefficients	t	*p*	95% Confidence interval		Collinearity Statistics				
		B	Std. Error	ß			Lower	Upper	Tolerance	VIF			
(Constant)		16.439	1.157		14.207	< 0.001	14.168	18.710			15.577	< 0.001	0.148
Age		− 0.002	0.011	− 0.009	− .223	0.824	− 0.024	0.019	0.695	1.439			
Sex (Female)		− 0.257	0.325	− 0.026	− .791	0.429	− 0.895	0.381	0.929	1.077			
Number of chronic diseases		0.044	0.088	0.018	.493	0.622	− 0.130	0.217	0.792	1.262			
Number of drugs used (Four and above)		0.970	0.351	0.103	2.765	**0.006**	0.281	1.658	0.738	1.355			
Anxiety		− 0.123	0.043	− 0.116	− 2.851	**0.004**	− 0.207	− 0.038	0.611	1.638			
Depression		− 0.068	0.049	− 0.064	− 1.407	0.160	− 0.164	0.027	0.491	2.035			
Social support		0.029	0.011	0.101	2.788	**0.005**	0.009	0.050	0.772	1.295			
Spiritual well-being	Meaning	0.096	0.089	0.056	1.081	0.280	− 0.079	0.271	0.376	2.657			
	Peace	− 0.009	0.089	− 0.005	− .101	0.920	− 0.184	0.166	0.346	2.889			
	Faith	0.247	0.045	0.222	5.433	** < 0.001**	0.158	0.336	0.608	1.644			

Bold indicates *p* < 0.05 statistical significance

## Dıscussıon

Medication adherence is an important factor for disease management in individuals with chronic diseases. Although there are many studies examining medication adherence in individuals with chronic diseases (Fan et al., 2021; Gonzalez Heredia et al., 2021; Kołtuniuk & Rosińczuk, 2021), no study has been found examining the factors associated with medication adherence within the scope of the biopsychosocial-spiritual model. This is a study that evaluates the correlations of biopsychosocial-spiritual factors on medication adherence in individuals with chronic diseases. The findings of this study showed that medication adherence in individuals with chronic diseases is more associated with psychological, social, and spiritual variables rather than biological variables such as age, gender, and number of chronic diseases. In the literature, it is stated that medication adherence is more associated with psychological state, social support, and spiritual status rather than biological variables such as age and gender (Abdul Wahab et al., 2021; Aldan et al., 2022).

The findings of this study showed that anxiety, one of the psychological variables, related to medication adherence. It is observed that the medication adherence levels of individuals with chronic diseases who experience high anxiety decrease. Surprisingly, this study found that depression had no significant association on medication adherence. A study of individuals with coronary heart disease showed that worsening anxiety symptoms led patients to become less likely to adhere to medical treatment and health practices (Fan et al., 2021). In a systematic review of individuals with type 2 diabetes, it was emphasized that anxiety and depression have a negative effect on patients’ medication adherence (Świątoniowska-Lonc et al., 2021). A study conducted with multiple sclerosis patients concluded that anxiety and depression had a significant negative impact on medication adherence (Kołtuniuk & Rosińczuk, 2021). A study conducted with individuals with type 2 diabetes showed that individuals experiencing anxiety were more likely to have medication non-adherence (Gonzalez Heredia et al., 2021). As anxiety increases, individuals become hopeless about their illness and their belief in treatment decreases, which may lead to decreased medication adherence.

Psychological variables were also associated with social support and spiritual state. In our study, it was determined that anxiety and depression levels of individuals with high perceptions of social support and good spiritual well-being decreased. Studies have shown that social support and spiritual well-being make a significant contribution to improving psychological status (Ratajska et al., 2020; Zhao et al., 2022). A study conducted with individuals with chronic diseases found that individuals with a high perception of family support had lower levels of depression and anxiety (Iovino et al., 2023). In light of all this information, it becomes clear that social support and spirituality should not be neglected in individuals with chronic diseases.

The findings of this study showed that social support has a relationship on medication adherence. It is observed that the medication adherence levels of individuals with chronic diseases with high social support levels increase. In a systematic review conducted on individuals with hypertension, it was determined that social support positively affected medication adherence (Shahin et al., 2021).

In a study conducted with individuals with chronic diseases, it was concluded that individuals with more social support had a higher level of medication adherence. Additionally, the study found that as the frequency of daily medication use increased, the medication adherence level of individuals decreased (Al-Noumani et al., 2023a, 2023b). In another study conducted with individuals with chronic diseases, it was determined that social support had a positive effect on medication adherence (Lu et al., 2020). In a qualitative study conducted with individuals with chronic diseases, it was found that individuals who received social support from their spouse, family, or friends had a higher level of adherence with the treatment regimen (Vahedparast et al., 2018). Social support provided to individuals with chronic diseases is very important for improving medication adherence and successful management of the disease (Shahin et al., 2021). Social support from family, friends, and other individuals can provide education to individuals with chronic diseases, support their behavior, monitor their medication intake, and improve medication adherence by providing reminders. In addition, social support can reduce the stress of individuals with chronic diseases, increase their self-confidence, and facilitate coping with the disease and medication adherence.

The findings of this study showed that faith has a relationship on medication adherence. It is observed that individuals with chronic diseases and high levels of faith have high levels of medication adherence. A study conducted on individuals with hypertension concluded that medication adherence increased as the level of spiritual well-being increased (Aşiret & Okatan, 2019). A study on patients with chronic obstructive pulmonary disease (COPD) concluded that individuals with higher spiritual well-being had higher medication adherence (Elhag et al., 2022; Helvaci et al., 2020). A study conducted in cancer patients concluded that there is a positive relationship between spiritual well-being and medication adherence, and that spiritual well-being has a mediating role between being religious and medication adherence (Shirinabadi Farahani et al., 2022). Spiritual well-being can affect individuals’ thoughts about their illness, the way they manage the illness, and their medication adherence. Therefore, evaluating the spiritual well-being levels of individuals with chronic diseases and integrating spiritual well-being interventions into the care plan may contribute to increasing the medication adherence level of individuals.

In a study conducted on patients with hypertension, it was determined that the level of spiritual well-being decreased as age increased (Aşiret & Okatan, 2019). In a study conducted with individuals with chronic diseases, it was concluded that the level of spiritual well-being decreases as age increases (Kütmeç Yılmaz & Kara, 2021). This situation can be explained by the importance of religion in the society where the study was conducted. People attach importance to religious values at every stage of their lives and shape their lives according to their religious values. However, this may be due to individuals experiencing functional losses that restrict their religious activities due to advanced age and multiple chronic diseases. Spiritual well-being can vary depending on many factors such as general health status, cultural characteristics, living conditions, personal characteristics, socioeconomic factors, and religious beliefs. The findings of this study showed that the level of spiritual well-being decreases as the number of chronic diseases increases. A study conducted on individuals with hypertension concluded that the spiritual well-being level of individuals with additional chronic diseases decreased (Aşiret & Okatan, 2019).

### Limitations and Strengths

This study has some limitations and strengths. First, the most important limitation of this study is that it is structured as an online survey study. This study was conducted online because it was aimed to reach individuals with chronic diseases from all over the country, but this reduced the possibility of individuals without internet access to access the survey. Other limitations of this study include the inclusion of people with access to a smartphone or computer and the decrease in smartphone use at older ages, which may have led to a lower average age. Another limitation of this study was that all scales were self-report; this can lead to the problem of self-report bias, where individuals may provide inaccurate or biased information about themselves. The Spiritual Well-Being Scale is widely used to assess spiritual well-being in medical research in Türkiye; however, the scale is intertwined with indicators such as meaning and purpose, peacefulness, harmony, strength, and comfort. The authors highlight that these elements overlap with mental health, potentially leading to misleading interpretations (Koenig & Carey, 2024, 2025). These limitations can affect the generalizability of the study.

One of the strengths of this study is its comprehensive approach, examining a wide range of biological, psychological, and social factors influencing medication adherence, providing a holistic perspective. Another strength is the large sample size (*n* = 837), which enhances the generalizability of the findings and increases statistical power.

### Implications for Healthcare Professionals

When evaluating medication adherence levels of individuals with chronic diseases, healthcare professionals should not forget that it is a multidimensional concept and should be evaluated together with psychological, social, and spiritual variables. Healthcare professionals can lead initiatives to develop and promote education and counseling services that are designed to empower patients and equip them with the knowledge and skills needed to manage their chronic conditions effectively. Structured education programs are a vital tool in this regard. Healthcare professionals can design and deliver these programs to address various barriers to adherence, such as lack of knowledge about medications, fears about side effects, or challenges in managing complex medication schedules. Social support is another critical area where healthcare professionals can make a significant impact. By involving family members, caregivers, and community resources, healthcare professionals can create a support network that encourages and reinforces medication adherence. Psychological interventions, such as stress management techniques, motivational interviewing, or cognitive-behavioral therapy, are additional areas where healthcare professionals can intervene effectively. Healthcare professionals can support patients by acknowledging and addressing their spiritual needs, offering resources such as chaplaincy services, or integrating spiritual practices into the care plan.

## Conclusıon

This study demonstrated that the biopsychosocial-spiritual model is a useful guide for assessing medication adherence in individuals with chronic diseases. Our study results showed that psychological, social, and spiritual factors have a relationship on medication adherence. Individuals with chronic diseases who experience less anxiety have higher levels of medication adherence. Additionally, individuals with improved perceptions of social support and spiritual well-being have higher levels of medication adherence. In the future, it is important to explore the impact of biopsychosocial and spiritual factors on medication adherence in studies with experimental designs.

## Data Availability

Data are available on request from the corresponding author.
