# Correction to: The Effect of Biopsychosocial-Spiritual Factors on Medication Adherence in Chronic Diseases in Türkiye

**DOI:** 10.1007/s10943-025-02396-2

**Published:** 2025-07-26

**Authors:** Aylin Bilgin, Ayser Doner, Gulyeter Erdogan Yuce, Gamze Muz

**Affiliations:** 1 Internal Medicine Nursing Department, Faculty of Health Sciences, Sakarya University of Applied Sciences, 54400 Sakarya, Turkey; 2 Department of Internal Medicine Nursing, Semra ve Vefa Kucuk Faculty of Health Sciences, Nevsehir Haci Bektas Veli University, Nevsehir, Turkey; 3 Department of Emergency Assistance and Disaster Management, Faculty of Health Sciences, Aksaray University, Aksaray, Turkey

**Correction to: Journal of Religion and Health** 10.1007/s10943-025-02317-3.

In this article, the title was incorrectly given as ‘The Effect of Biopsychosocial-Spiritual Factors on Medication Adherence in Chronic Diseases in Turkey Using Structural Equation Modeling’ but should have been ‘The Effect of Biopsychosocial-Spiritual Factors on Medication Adherence in Chronic Diseases in Türkiye’.

The original article has been corrected.

